# Nanotribological behavior of deep cryogenically treated martensitic stainless steel

**DOI:** 10.3762/bjnano.8.177

**Published:** 2017-08-25

**Authors:** Germán Prieto, Konstantinos D Bakoglidis, Walter R Tuckart, Esteban Broitman

**Affiliations:** 1 Grupo de Tribología, Departamento de Ingeniería, Universidad Nacional del Sur, Bahía Blanca, Buenos Aires, Argentina; 2 Consejo Nacional de Investigaciones Científicas y Técnicas, CABA, Argentina; 3 IFM, Linköping University, SE581 83 Linköping, Sweden

**Keywords:** carbide refinement, cryogenic treatments, friction, nanoindentation, nanoscratch, wear-resistance improvement

## Abstract

Cryogenic treatments are increasingly used to improve the wear resistance of various steel alloys by means of transformation of retained austenite, deformation of virgin martensite and carbide refinement. In this work the nanotribological behavior and mechanical properties at the nano-scale of cryogenically and conventionally treated AISI 420 martensitic stainless steel were evaluated. Conventionally treated specimens were subjected to quenching and annealing, while the deep cryogenically treated samples were quenched, soaked in liquid nitrogen for 2 h and annealed. The elastic–plastic parameters of the materials were assessed by nanoindentation tests under displacement control, while the friction behavior and wear rate were evaluated by a nanoscratch testing methodology that it is used for the first time in steels. It was found that cryogenic treatments increased both hardness and elastic limit of a low-carbon martensitic stainless steel, while its tribological performance was enhanced marginally.

## Introduction

AISI 420 is a martensitic stainless steel, commonly used in pumping applications in the petrochemical industry, oil extraction and energy generation. As a result, components made of AISI 420 are subjected to severe mechanical and tribological solicitations. Therefore, the enhancement of wear resistance of this steel is of technological and industrial interest.

Although enhancing the wear resistance of steel alloys by means of cryogenic processing has been known since at least the last three decades [1], the metallurgical phenomena responsible for this modifications are still under discussion. The main operative mechanisms during the cryogenic treatment of steels discussed in the current state-of-the-art literature are: transformation of retained austenite [2–4], carbide refinement [5–7] and plastic deformation of virgin martensite [8–9].

Because of the high hardenability of low-carbon AISI 420 stainless steel, both transformation of retained austenite and plastic deformation of virgin martensite do not seem to be operative during cryogenic cooling. The main effect observed in AISI 420 after cryogenic processing is a strong reduction in carbide size and a more even dispersion of them in the martensitic matrix [10]. This carbide refinement improved the macroscale wear resistance of the material by 35% under lubricated and by 90% under dry sliding conditions compared to the conventionally treated specimens [11]. A slight reduction in the macroscale friction coefficient has also been observed [11] and an increase in fracture toughness (ca. 30%) was reported in deep cryogenically treated specimens [12].

Nanotribological tests, such as the nanoscratch technique described in [13], can be used for studying the influence of microstructural features on the frictional and wear behavior of a material, thanks to the small size of the tip counterpart, and applied loads in the order of micronewtons. A nanotribological approach is necessary, as most engineering surfaces begin to contact at the tip of asperities, whose dimensions are at the nanometric scale. However, so far the majority of the research efforts available in the open literature were focused on the micro- and nanotribological evaluation of Si and single metals for microelectromechanical system (MEMS) applications [14–15] and carbon-based coatings [16–18]. Although, there is an incipient amount of nanotribological studies performed in engineering steels [19–24].

Tribometers built on nanoindentation-based equipments, such as triboindenters, made possible the study of friction and wear at small contact scales. This kind of equipment can be used to simulate a single sharp asperity sliding over a surface while simultaneously controlling with high precision the applied force, and measuring the topographical modifications and the friction forces.

The purpose of this paper is to deepen the understanding of the influence of cryogenic treatments on the wear resistance and the mechanical properties of a low-carbon AISI 420 martensitic stainless steel evaluated at very small scales.

## Experimental

The material used in this study was a low-carbon AISI 420 martensitic stainless steel. Its chemical composition is presented in Table 1 and was determined using an Spectro SPECTROMAXx optical emission spectrometer.

**Table 1:** Chemical composition of AISI 420.

element	C	Cr	Mn	Si	P	S	Fe

content in AISI 420 (wt %)	0.17	12.83	0.76	0.55	0.05	0.017	balance

**Figure 1:**
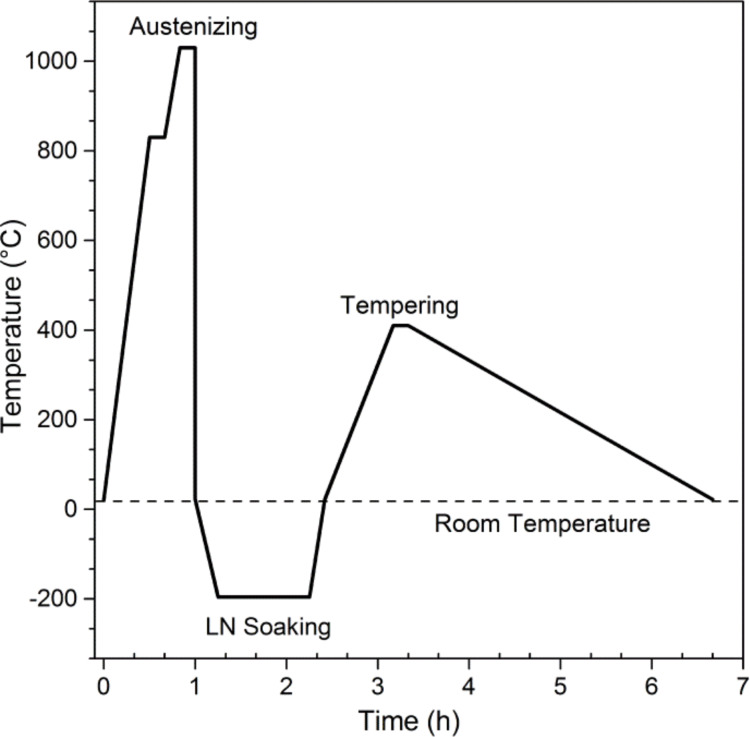
Representation of the applied cryogenic treatment.

AISI 420 specimens were pre-heated at 830 °C for 10 min, followed by quenching in oil from 1030 °C, and afterwards annealed at 410 °C for 10 min with furnace cooling. This group was identified as conventionally heat-treated (CHT). The other group of specimens was quenched in oil from 1030 °C and immediately afterwards soaked in liquid nitrogen, at an equilibrium temperature of −196 °C. The cooling rate was set at 0.45 °C/s and the soaking time at cryogenic temperature was of 2 h. Finally, the specimens were annealed at 410 °C for 10 min and slowly cooled inside the furnace (Figure 1). This latter group was identified as deep cryogenically treated (DCT). The austenization, quenching and annealing of the specimens was performed in argon atmosphere in order to prevent decarburation. The selection of the heat-treatment parameters was based in the results of our previous work [10].

Scanning electron microscopy (SEM) was used to characterize the resulting microstructures after each heat treatment (JEOL JSM-35CF). The volume fraction of carbides was estimated from the SEM micrographs using Delesse’s principle for stereographic relationships [25].

All specimens were included in carbon-filled bakelite using a Buehler SIMPLIMET 3 hot press, and then were polished with a Struers TEGRAMIN-30 automatic polishing machine, employing diamond suspensions down to 0.25 μm in particle size. The obtained roughness parameters, measured with a Hysitron TI950 triboindenter, were: *R*_a_ = 15 ± 3.2 nm, *R*_z_ = 98 ± 18 nm, *R*_t_ = 125 ± 31 nm. The polished specimens were ultrasonically cleaned with acetone and isopropyl alcohol for 5 min and then placed onto the stage of the triboindenter to perform nanoindentation and nanotribological tests.

### Nanoindentation tests

A Hysitron TI950 triboindenter was employed for performing nanoindentation measurements, using a three-plate capacitive transducer. This transducer can act both as the actuator and the sensing device and allows for the application of normal forces up to 10 mN. The triboindenter utilizes the Oliver and Pharr (O&P) method [26] as the standard procedure to interpret the data from nanoindentations. According to [26], hardness (*H*) is defined as:

[1]
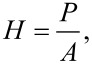


where *P* is the maximum normal load and *A* is the contact area between the tip and the specimen. The contact area can be related to the contact stiffness by using Sneddon’s law [27]:

[2]
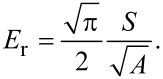


Nanoindentation tests were performed using a Berkovich diamond tip, with an apex radius of ca. 100 nm. Penetration depths of 50, 100, and 200 nm were set. A 3 × 4 array of indentations was performed in the specimens at each penetration depth, spaced at 20 μm from each other. The significance of the obtained results was determined by analysis of variance (ANOVA), using the statistical software INFOSTAT [28].

When carrying out nanoindentations in an elastic–plastic material like a metal, it tends to accumulate around the indenter, forming a pile-up that is higher than the sample surface. This phenomenon can lead to the underestimation of the true contact area and a significant deviation of calculated hardness and elastic modulus from their real values. The formation of pile-ups during nanoindentation of steels has been studied by several researchers [29–31].

Our approach was to compare the conventional O&P method with the one proposed by Joslin and Oliver (J&O) [32]. The J&O method utilizes the ratio between the hardness and the square of the elastic modulus (*H*/*E^2^*) as an independent characteristic parameter. The proposed method utilizes the maximum force applied during the test (*P*) and the calculated contact stiffness (*S*) from the nanoindentation data. *S* is defined as the slope of the unloading curve (∂*P/∂h*), evaluated at the point of maximum force. Both *P* and *S* and can be determined without knowledge of the exact geometry of the diamond tip or the shape and size of the indentation. The values of *P* and *S* are related through the following equation [32]:

[3]
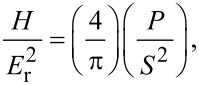


where *E*_r_ is the relative elastic modulus, defined as

[4]
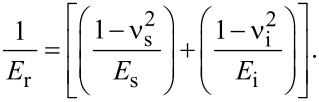


*E*_s_ and ν_s_ are Young’s modulus and Poisson’s ratio of the sample, and *E*_i_ and ν_i_ are Young’s modulus and Poisson’s ratio of the indenter (*E*_i_ = 1140 GPa, ν_i_ = 0.07).

This approach does not allow for the simultaneous determination of *E* and *H*, but several researchers [33–34] have reported *P*/*S*^2^ (i.e., *H*/*E*_r_^2^) as a useful characterizing parameter, even when the development of pile-up is considerable [35].

### Nanotribology tests

**Figure 2:**
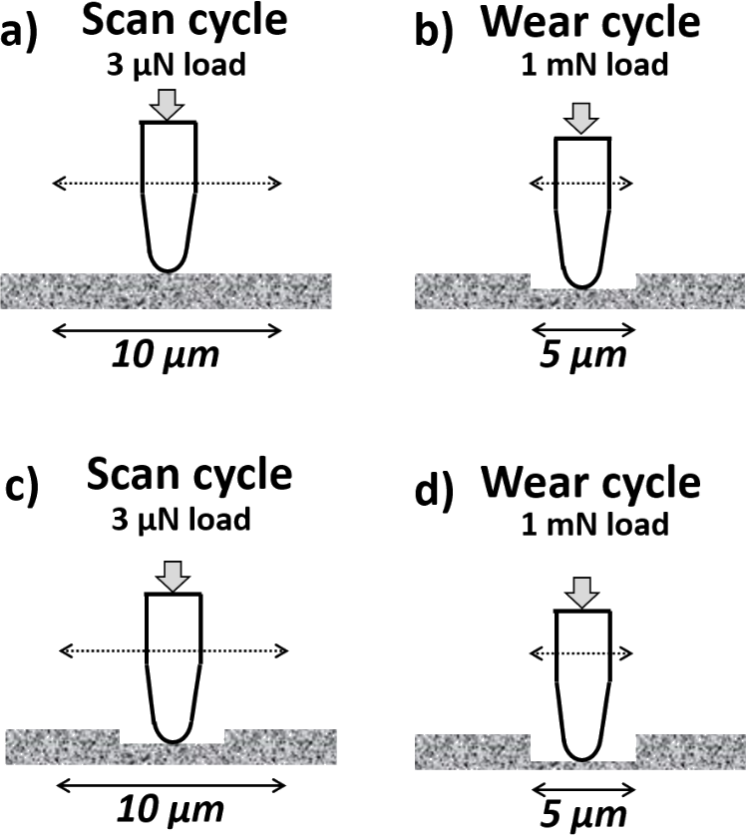
Schematics of the used scratch method to measure friction and wear: (a) pre-scan to get the initial topographical profile; (b) first cycle of wear; (c) post-scan to get the new topographical profile; (d) three cycles of wear. The processes (c) and (d) are repeated ten times. Reprinted with permission from [13], copyright 2015 AIP Publishing LLC.

In order to evaluate the frictional behavior of the samples and their wear resistance, microscale friction tests were performed. The experimental setup followed the guidelines of the test procedure proposed by Broitman and Flores [13]. In this method, a probe is continuously scanning a track in a reciprocal movement, as shown in Figure 2. In our work, a 1 mN load was applied in a stroke length of 5 µm for 31 cycles to evaluate the evolution of the friction coefficient, and a load of 3 µN in a stroke of 10 µm for 12 scanning cycles was used to evaluate the surface roughness. The obtained topographic information at the low load is used to calculate the wear rate and roughness evolution, while the force transducers measure the friction force variations at the higher applied loads. The method utilizes a MatLab^®^ script to eliminate the thermal drift. The software output gives the resulting friction coefficient, track roughness, and wear rate as a function of the number of cycles of the probe. The wear volume is estimated considering the projected area *A* of the tip as a function of the penetration depth (*h*): *A* = (*2Rh* − *h*^2^), where *R* is the tip radius. The volume of the displaced material during each cycle is calculated as the sum of areas at the different penetration depths of the track. Additionally, the evolution of the average trench roughness (*R*_a_) is calculated after every cycle as 100 × *R*_a_/*R*_0_, where *R*_0_ is the average roughness before the first test cycle. It should be pointed out that, in this method, the wear is calculated after the elastic recovery of the surface took place.

For the nanoscratch tests, a conical diamond tip with an apex radius of 5 μm was employed. The applied normal load was set at 1000 μN and each reported value corresponds to the average of at least three valid tests. The theoretical Hertzian contact pressure was estimated at 10.7 GPa.

In order to evaluate if the scratch tests were generating wear and not only plastically deforming the surfaces, the elastic recovery (ER) was calculated for each sample by nanoindentation using the same tip and normal load as in the scratch tests. ER is calculated as the ratio between the maximum (*h*_t_) and the residual (*h*_r_) height as follows:

[5]
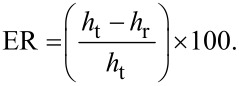


## Results and Discussion

### Microstructural characterization

**Figure 3:**
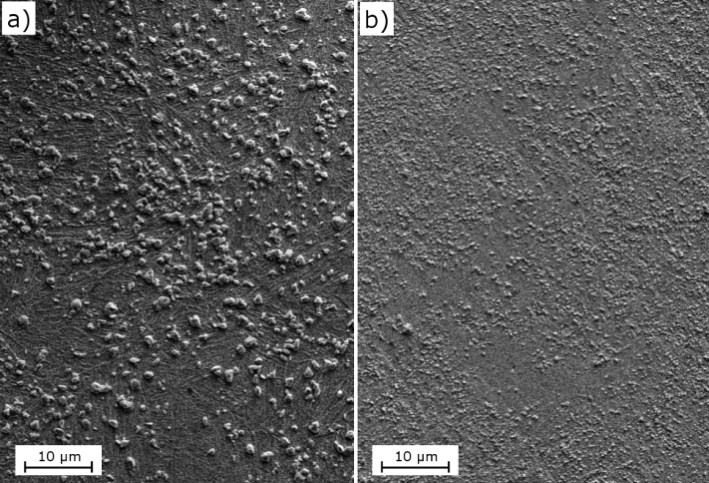
SEM image of a) CHT specimen and b) DCT specimen, showing a martenstic matrix with precipitated globular carbides.

SEM micrographs of the specimens after the heat treatments are shown in Figure 3. It can be seen that in both cases the microstructure consisted of a martensitic matrix with precipitated globular carbides. The application of the cryogenic treatment generated a strong reduction in the size of carbides and an increase in the amount of particles, changing the mean carbide diameter of 0.9 μm for CHT specimens to 0.4 μm in DCT samples. The volume fraction of carbides was estimated to be 16.8% in CHT specimens, whereas for the DCT ones it was 11.9%. Similar reductions of the carbide size were reported by Das and co-workers [3,36].

### Nanoindentation tests

A summary of the results from the nanoindentation tests performed with the Berkovich tip is shown in Table 2. It can be seen that the residual height (*h*_r_) was smaller for DCT specimens at all penetration depths, meaning that they had a larger amount of elastic recovery during unloading also depicted by the higher values of the *h*_r_/*h*_t_ ratios. The ANOVA test indicated that these differences were statistically significant. Furthermore, the ANOVA analysis of the maximum applied load (*P*_max_) for each penetration depth has shown no statistically significant differences for both CHT and DCT specimens. According to Bolshakov and Pharr [37], the *h*_r_/*h*_t_ ratios are in all cases at the limit of applicability of the O&P method, i.e., above ca. 0.7 in materials that develop pile-ups. The maximum penetration depth has been predefined by the displacement control condition, and the force required to reach each depth was the same for both groups of specimens. Hence, the DCT samples must have a higher elastic limit [38–39].

**Table 2:** Summary of nanoindentation results.

penetration depth (nm)	residual height (nm)		maximum force (μN)		*h*_r_/*h*_t_		contact stiffness (μN/nm)	
	CHT	DCT	CHT	DCT	CHT	DCT	CHT	DCT

50	34.5 ± 0.9	32.6 ± 1.2	887 ± 51	877 ± 48	0.651	0.690	75.6 ± 1.8	65.1 ± 1.7
100	74.6 ± 1.7	71.8 ± 1.2	2122 ± 137	2153 ± 99	0.719	0.746	108.7 ± 2.3	87.9 ± 2.4
200	153.6 ± 1.9	145.0 ± 2.7	5788 ± 259	5983 ± 276	0.725	0.768	160.9 ± 2.4	126.9 ± 1.8

The aforementioned phenomenon can be seen more clearly from the analysis of the contact stiffness, as DCT specimens showed significantly smaller values of contact stiffness (*S*) at all penetration depths. If we assume that the elastic modulus does not change with the application of cryogenic treatments, then it follows from Equation 2 that the true contact area between the indenter and the specimen has to be smaller in the DCT samples. This smaller contact area can be accounted by a higher amount of elastic recovery (as evidenced by the residual height) and also by the formation of smaller pile-ups.

In order to characterize the resistance of the material to plastic deformation, the parameter *H*/*E*_r_^2^ has been calculated by two different approaches (Table 3). It can be seen that there is no significant difference between the methods, which could mean that pile-up is not so severe, at least at penetration depth of 50 and 100 nm. At 200 nm, pile-up influence shows a marked increase, as *h*_f_/*h*_max_ is higher than 0.7. However, the values from Table 3 are useful for comparison purposes between CHT and DCT specimens. The values of *H*/*E*_r_^2^ for cryogenically treated specimens were ca. 30% higher than those of the conventionally treated samples at penetration depths of 50 and 100 nm, independently of the calculation method. At 200 nm, this difference is 56% when applying the O&P method and 67% with the J&O method. These results also support the hypothesis that the cryogenic treatment increased the elastic limit of the specimens.

**Table 3:** Comparison of the *H*/*E*_r_^2^ values obtained by the Oliver & Pharr and Joslin & Oliver methods.

penetration depth (nm)	*H*/*E*_r_^2^ (GPa^−1^ × 10^−4^)			
	Oliver & Pharr		Joslin & Oliver	
	CHT	DCT	CHT	DCT

50	2.12 ± 0.10	2.82 ± 0.19	1.98 ± 0.10	2.64 ± 0.18
100	2.45 ± 0.13	3.15 ± 0.16	2.29 ± 0.12	2.95 ± 0.15
200	3.14 ± 0.11	4.92 ± 0.24	2.85 ± 0.14	4.75 ± 0.26

The reduction of the carbide volume fraction in DCT specimens can be associated to a higher amount of undissolved carbon in the martensitic matrix. In addition, cryogenic treatments also increase residual stresses in the martensitic matrix, as we were able to measure in our previous work using X-ray diffractometry [10]. These residual stresses can be associated to a higher dislocation density, which in turn has been identified by Kehoe and Kelly [40] as the main factor affecting the strength of martensite materials with equal amounts of carbon. Due to the small scale of the test, the analysis primarily yields information regarding the metallic matrix. As carbides are much harder than martensite, it can be expected that they would sink into the matrix if they are hit by the diamond tip. As a result, the measured stiffness will be slightly higher than during indenting a “pure” matrix. The variations that we observe in the values of *H*/*E*_r_^2^ are then the result of hitting areas where the carbide is closer or further than in the other points.

With respect to the possible influence of the native oxide films, stainless steels develop oxide films of ca. 2 nm in thickness [41–42], thus its influence can be neglected as the penetration depths are much larger.

### Nanoscratch tests

**Figure 4:**
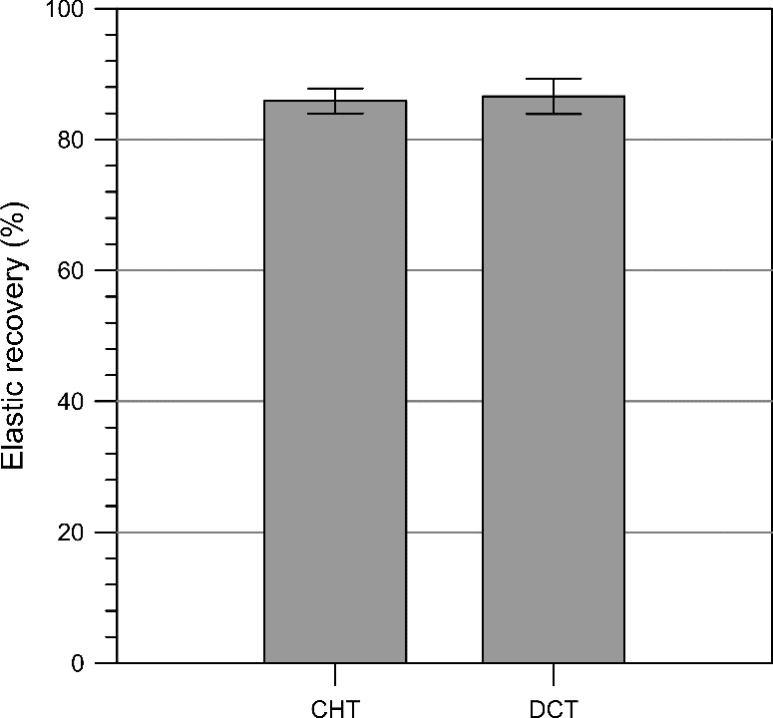
Elastic recovery values for nanoindentations performed with a conical indenter (*r* ≈ 100 µm) at 1000 µN of normal load.

Prior to the execution of the nanoscratch tests, we performed indentations with the conical indenter (apex radius ca. 100 µm) at the same normal load (1000 µN) that we used in the wear cycles. In Figure 4 it can be seen that the elastic recoveries for all samples was above 85% and that there was no significant difference between CHT and DCT specimens. These high values of elastic recovery are useful in order to evaluate whether we are effectively removing material during the nanoscratch test or whether we are plastically deforming the surface.

**Figure 5:**
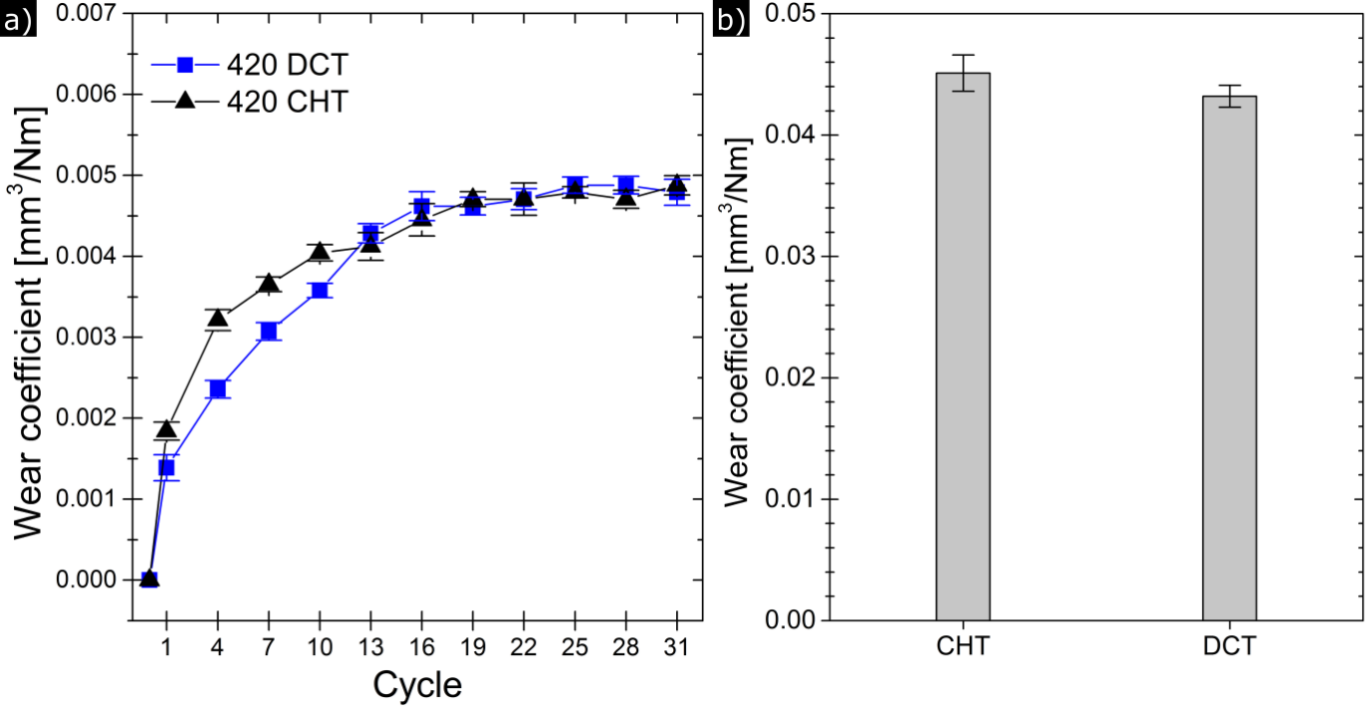
a) Evolution of wear coefficient during the tests at 1000 μN of applied normal load and b) cumulative wear coefficient.

Figure 5 shows the evolution of the wear coefficient during a complete run of the wear test. It can be seen that cryogenically treated specimens exhibited marginally lower wear rates during the initial stage of the tests.

**Figure 6:**
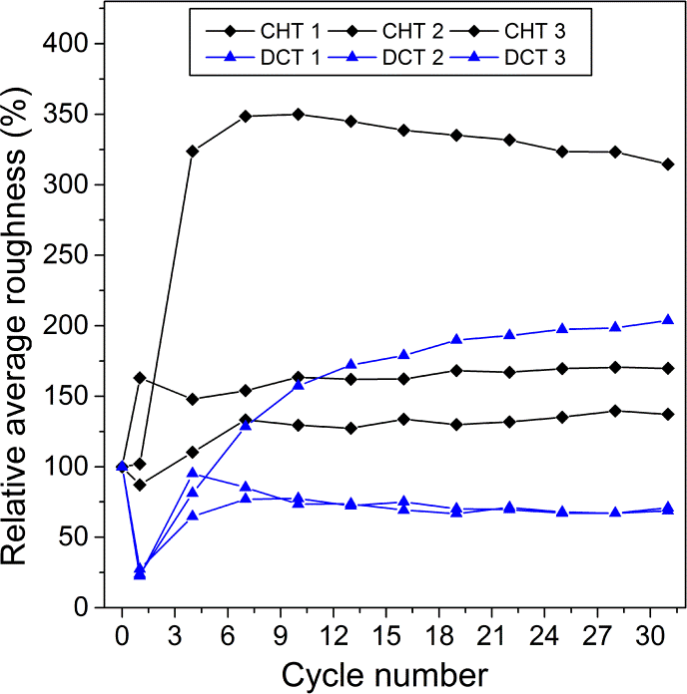
Evolution of the relative average roughness after each test cycle.

Cryogenically treated specimens had a lower amount of total wear (Figure 5b), although this difference was only marginal. Figure 6 shows the evolution of roughness for three complete test runs of each type of specimens. It can be seen that DCT specimens had a lower average roughness, and in two runs roughness reached a steady state after running-in, while for the CHT specimens, the first two runs showed an increasing trend and the third one had a marked increase (over 300%) from the initial roughness and a slight reduction afterwards. These differences in the roughness evolution could be associated to the modification of the elastic limit of the cryogenically treated specimens. Again, the effect of the oxide films can be neglected as they wear out after two or three cycles.

As a nanoscratch test is essentially an abrasion test using a single asperity, our results confirm that the increase in hardness shown by the DCT specimens led to a higher wear resistance following the classical approach of Rabinowicz [43].

**Figure 7:**
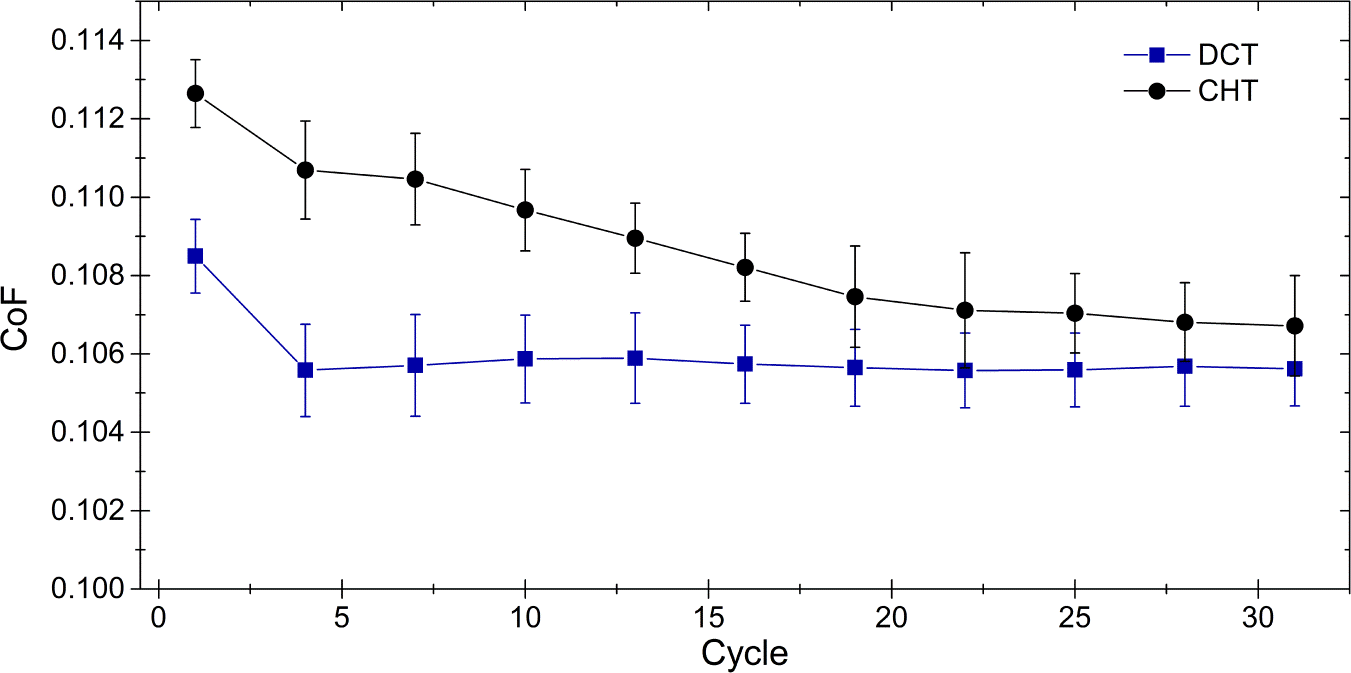
Evolution of the friction coefficient during the nanowear test.

**Figure 8:**
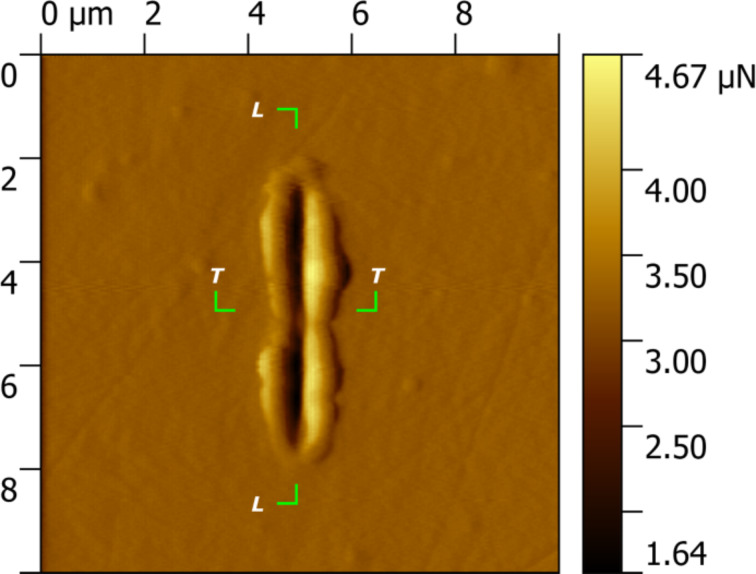
Scanning probe microscopy (SPM) image after 30 cycles of nanoscratch testing in a DCT specimen. The depth profiles below in Figure 9 were taken in the longitudinal (L–L) and transversal (T–T) directions.

Regarding the evolution of the friction coefficient (CoF), Figure 7 shows that it slightly diminished towards the end of the tests, mainly due to wear and deformation of asperities of the surface of the track (Figure 8) [44]. The level of friction reduction was between 2 and 5%. Similarly to the behavior of the wear coefficient, DCT specimens performed better than CHT samples. This difference was more pronounced during the initial passes of the test. However, the improvement of the friction behavior for DCT specimens should be considered marginal.

The evolution of the wear and friction coefficients (Figure 5 and Figure 7) shows that DCT specimens exhibit a marginally improved tribological behavior, i.e., less friction and wear than CHT specimens during the first passes of the diamond tip. We attribute this behavior mainly to the increased hardness and elastic limit of the martensitic matrix. Similarly, Xie et al. [45] has reported that a hardness increase implies a higher elastic shakedown limit of the material and a reduction of the friction coefficient.

Figure 8 shows a scanning probe microscopy (SPM) image of a DCT specimen after a nanoscratch test, where the formation of the wear track can be clearly seen. Material pile-up is visible at both sides of the trench, as well as at the entry and exit edges. The wear scar presents the typical features of an abrasion test performed in a ductile material.

**Figure 9:**
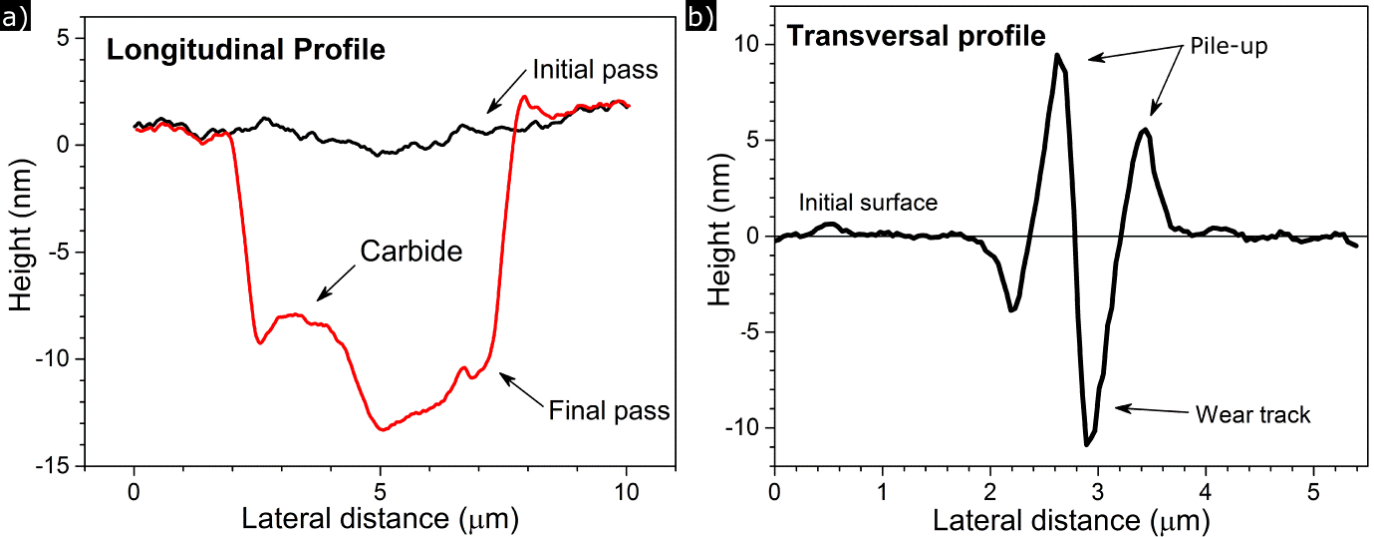
Depth profiles after a wear test in a DCT specimen in the a) longitudinal and b) transversal directions. The formation of the wear groove and the pile-ups around the track can be seen, as well as the probable revelation of a subsuperficial carbide.

Figure 9a presents the initial (black line) and the final (red line) longitudinal profiles of a DCT specimen, showing the formation of the trench. The total wear volume is the result of the combined adhesion, ploughing effect and cutting effects during the sliding process. The final profile of the scar reveals the probable presence of a subsuperficial carbide (Figure 9a), which did not wear as much as the metallic matrix due to its higher hardness. In the transversal profile (Figure 9b), material pile-up at the edges of the track can be clearly seen, in agreement with the plastic deformation observed in the nanoindentation tests.

It is interesting to compare the nanoscratch results with those from macroscopic tests reported by Prieto and Tuckart [11]. In that work, wear occurred mainly by delamination, driven by ratcheting, while in this present work wear is mainly abrasive. In [11], the smaller carbides in DCT specimens delayed the subsuperficial cracking due to a reduction in the stress concentration effect. Instead, in this present work wear tests were reciprocal, therefore ratcheting was not operative and the carbides played a secondary role in the wear response of the material. This was also a consequence of the scale of the wear tracks, which we infer were of the order of the distance between carbides.

The combination of nanoindentation and nanoscratch tests allowed us to have a better understanding of the role of the martensitic matrix and its contribution to the wear resistance of the material. This contribution could not be analyzed in the macroscopic tests performed in [11] due to the large scale of the tribological interactions and the type of sliding conditions.

## Conclusion

Considering the results obtained from the study of the mechanical and tribological properties of a cryogenically treated martensitic AISI 420 stainless steel, we conclude that cryogenically treated specimens show a higher amount of undissolved carbon in the martensitic matrix, therefore leading to an increased hardness and elastic limit in comparison with the conventionally treated ones.

The carbide refinement developed in cryogenically treated specimens had a marginal contribution in preventing abrasive wear at the small scale of our test. Instead, we propose that the improved mechanical resistance of the cryogenically treated martensite was responsible for the reduction in friction and the marginal decrease in the wear coefficient.
